# Angiosarcoma of the Maxillary Sinus: A Case Report

**DOI:** 10.7759/cureus.63131

**Published:** 2024-06-25

**Authors:** Gowtham Narasimhan, Prasad T Deshmuk, Sagar S Gaurkar, Farhat Q Khan

**Affiliations:** 1 Otolaryngology, Jawaharlal Nehru Medical College, Datta Meghe Institute of Higher Education and Research, Wardha, IND; 2 General Surgery, Jawaharlal Nehru Medical College, Datta Meghe Institute of Higher Education and Research, Wardha, IND

**Keywords:** nasal soft-tissue mass, nasal angiosarcoma, spindle cell, paranasal sinuses, angiosarcoma

## Abstract

Angiosarcoma can be defined as a malignant neoplasm arising from the lining of the blood and lymphatic vessels, including the endothelial cells. It can occur in any body part, such as blood vessels, skin, liver, and breast. Its incidence varies based on the site. There are different underlying etiologies associated with the incidence of angiosarcoma. Clinical presentation depends on the site of origin. Angiosarcoma of the sinus or nasal openings can be observed as a tissue mass, lesions, obstructed nasal cavity, facial swelling, proptosis, anosmia, nasal discharge, and epistaxis. These are rare malignancies with very low incidence. Though it has been reported in all age groups, it is more common in adults in their sixth decade and more. Nasal angiosarcoma can be a diagnostic challenge due to its rarity. This is a case of a 56-year-old female with a major complaint of nasal obstruction and face swelling for two months. Physical examination revealed a pinkish polypoidal mass. A contrast-enhanced computed tomography scan showed a heterogeneously arterial enhancing soft-tissue lesion in the left maxillary sinus with significant erosive changes. Histopathological analysis revealed a malignant spindle cell tumor, which was confirmed by a CD34 immunohistology stain. The patient was advised surgical excision for further management, which was denied. The patient is undergoing radiation therapy and is on third cycle as per the last follow-up.

## Introduction

Angiosarcomas are aggressive malignancies arising from the mesenchymal cells with more than 50 subtypes of histopathological variants [1]. Angiosarcomas are vascular phenotypic malignant neoplasms with endothelial-like characteristics in their tumor cells, with high frequency in the neck and the head region in patients without any medical history [2]. Angiosarcoma of the nasal sinus is a rare presentation in paranasal and nasal sinuses, with a small incidence of approximately 0.1% [3]. Diagnosis of nasal angiosarcoma remains a diagnostic challenge, attributed to its low incidence and mild symptoms in the initial stage, which might result in a poor prognosis [2]. Clinical presentation of nasal sarcomas is usually associated with airway obstruction, facial swelling, and proptosis with or without epistaxis [4]. Angiosarcomas frequently occur in any region of the body, including the forehead, scalp, nose, cheek, and neck. While nasal angiosarcoma cases are rare in English medical literature, there has been an increase in their incidence over the past decade [3]. Surgical excision remains the recommended treatment with or without skin grafts, though some cases have been treated with radiotherapy [5,6].

## Case presentation

**Figure 1:**
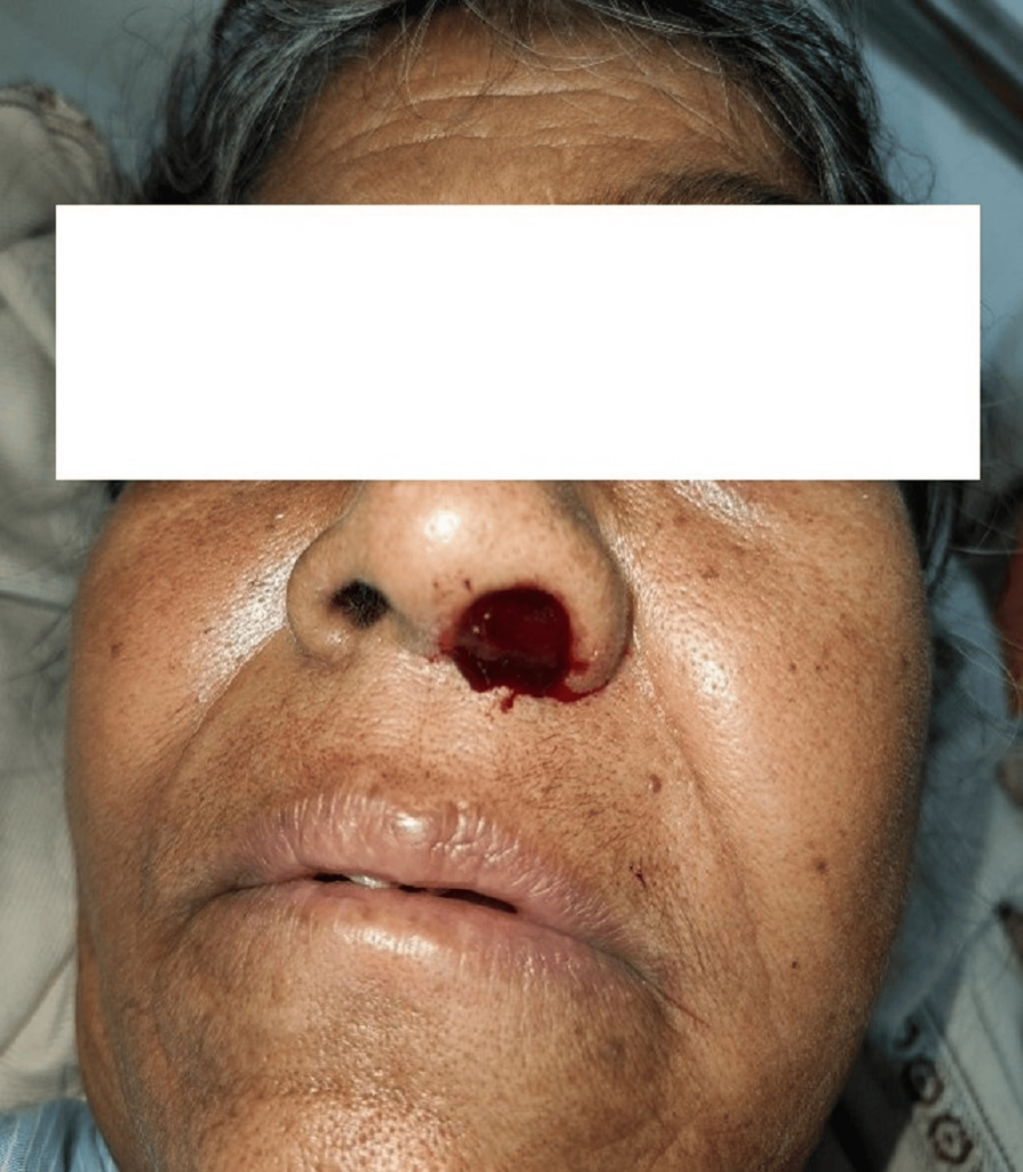
Physical presentation of the patient

A 56-year-old female presented to the outpatient department of our tertiary-care center in Maharashtra, India, with major complaints of left-side nasal obstruction and left-side facial swelling for two months. There was no history of radiation exposure, toxins, or related trauma, as well as any skin lesions. The patient had a history of systemic hypertension and diabetes for the last 13 years. Figure 1 shows the physical presentation of the nose and left side of the face.

**Figure 2:**
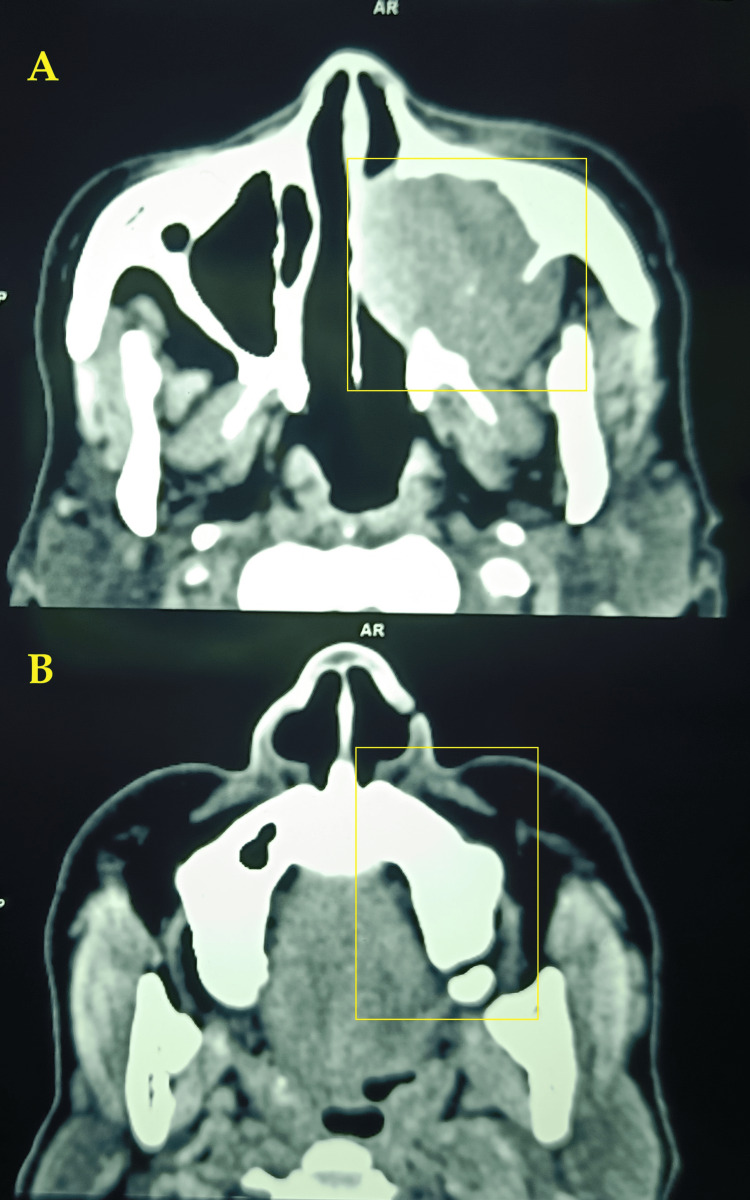
Contrast-enhanced computed tomography scan of (A,B) axial cuts showing the lesion in the left maxillary sinus extension into the left nasal cavity

Diagnostic nasal endoscopy revealed a 1.5 x 1.0 cm pinkish mass in the osteomeatal complex on the left side. Inferior and middle turbinate could not be differentiated separately. There was no lymph node enlargement in the neck. A contrast-enhanced computed tomography scan showed well-defined significantly heterogeneous arterial-enhancing soft tissue, as seen with the epicenter in the left maxillary sinus. Few calcific foci were seen within the lesion. The left maxillary sinus exhibits significant erosive changes in the medial, posterolateral, and superior walls (Figure 2).

**Figure 3:**
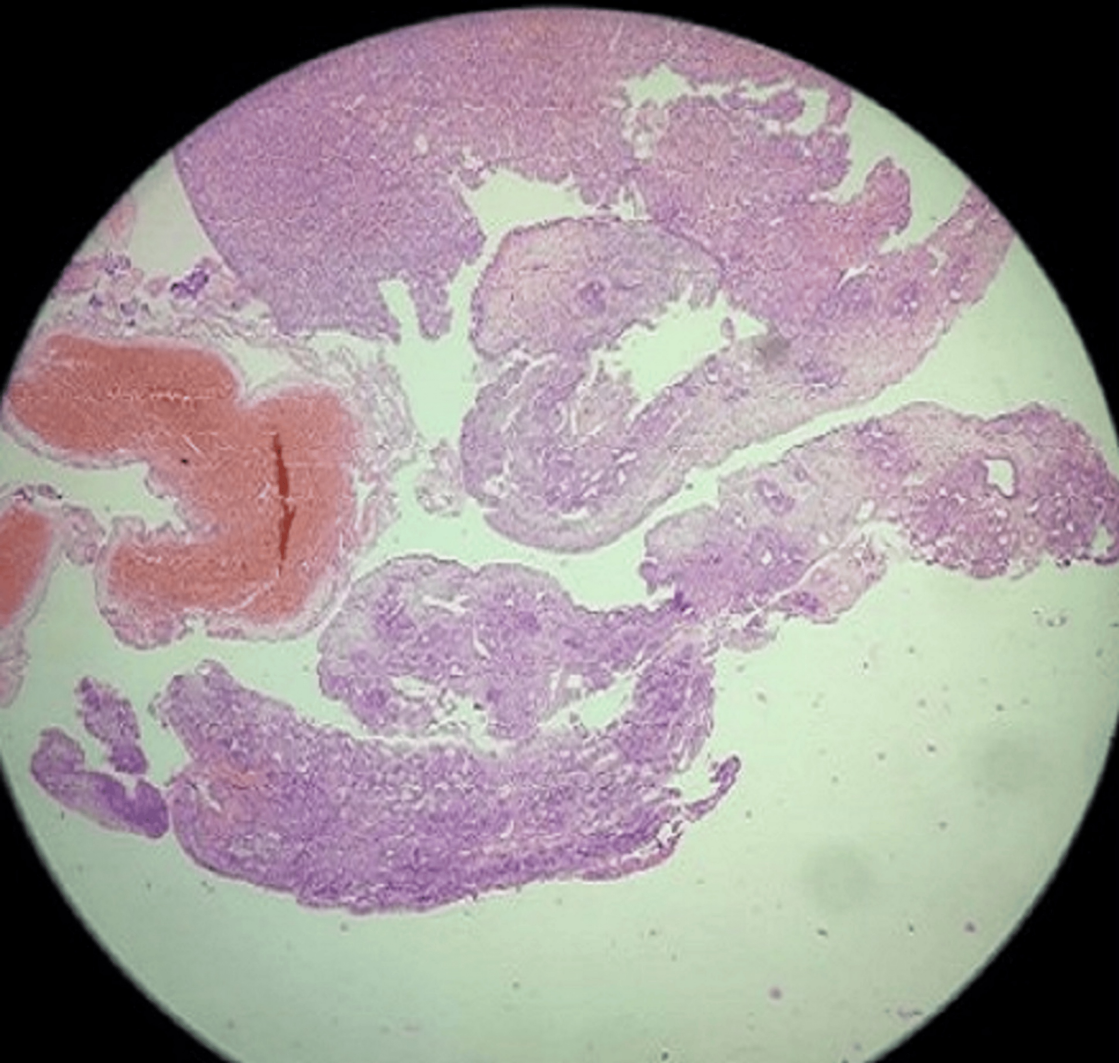
Hematoxylin and eosin staining of the excised specimen at 10× resolution

**Figure 4:**
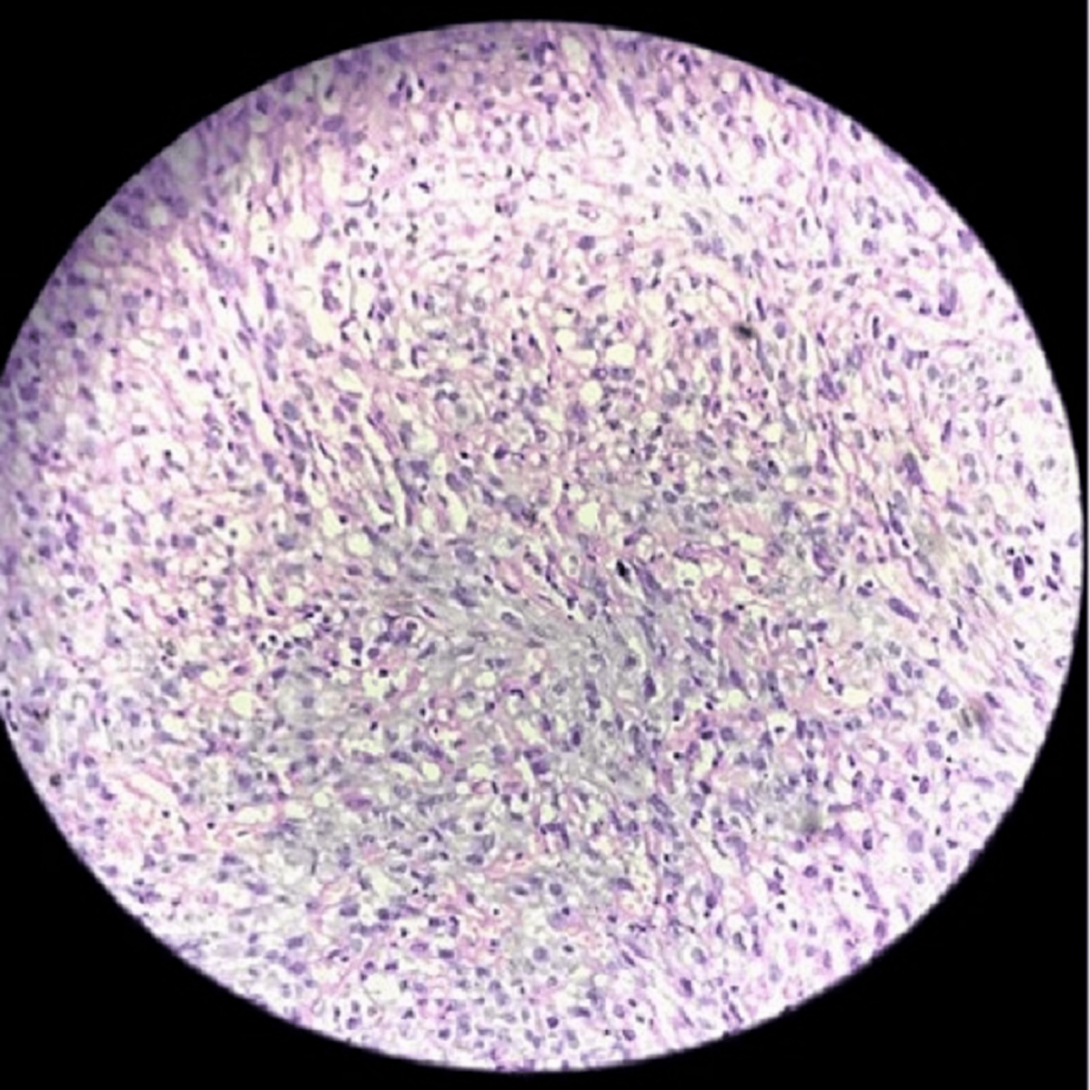
Hematoxylin and eosin staining of the excised specimen at 40× resolution

The patient was further subjected to endoscopic examination, and the excised specimen was sent for histopathological examination, which was suggestive of the malignant spindle cell tumor angiosarcoma. The microscopic view revealed small bits of respiratory epithelial lining. Submucosal areas show spindle cell proliferation arranged in fascicles and sheets, and ill-circumscribed infiltrating mass (Figures 3, 4).

On immunohistochemistry, the tumor cells showed diffuse strong cytoplasmic membrane positivity for CD34 and factor 8. The patient was advised to undergo a total maxillectomy but declined the surgery and was referred for radiotherapy sessions. The patient received definitive radiotherapy using the conventional three-field technique, with a total dose of 65 Gy in 30 fractions during a six-week period. The patient was advised to follow-up every two months.

## Discussion

Clinical presentation of the angiosarcoma is usually mild during the initial presentation. Nasal angiosarcomas are observed as lesions with irregular and ulcerated margins [5]. In the study by Chai et al. [2], a 40-year-old male presented with similar symptoms to this case, along with face numbness. Magnetic resonance imaging and computed tomography are found to be helpful in the diagnosis of these lesions, which can be further aided by immunohistochemical staining [3,4]. Potential differential diagnoses include hemangioma, Kaposi sarcoma, hemangioendothelioma, hemangiopericytoma, and other hemorrhagic conditions. Histopathological examination of the specimen demonstrated ill-formed vascular channels, and the cells show a high nuclear-cytoplasmic ratio with small amounts of clear to eosinophilic cytoplasm. Nuclei vary from spindle to oval to round. There is focal mitotic activity. On immunohistochemistry, the tumor cells showed diffuse strong cytoplasmic membrane positivity for CD34 and factor 8. The etiology of these tumors remains unclear but is associated with factors such as vinyl chloride exposure, chronic lymphedema, trauma, telangiectatic skin lesions, and radiotherapy. Angiosarcoma has been observed in the neck, head, scalp, cheek, forehead, mandible, and ethmoid sinuses. Diagnosis can be made by histopathological staining, which can be further confirmed by immunohistochemistry [1,3,4]. Due to its rarity, there are no standard recommendations for treating angiosarcoma in the nasal and paranasal sinus. Published research suggests using surgical excision and radiotherapy as the optimal treatments [6,7]. Angiosarcoma of the skin or soft tissue of the head and neck is associated with a 50% mortality rate within the first 25 months and a 12% survival rate at five years, compared to nasal cavity or paranasal sinus angiosarcoma, which has a 22% survival rate at five years according to the grade of differentiation and early diagnosis [7]. It is a rare and aggressive type with poor prognosis and suboptimal treatment options. Hence, skin angiosarcoma can be concluded to be more aggressive than nasal and paranasal sinus angiosarcoma.

## Conclusions

Nasal angiosarcoma is rare and aggressive. Though there are major advances in diagnostic modalities such as radiological and histological staining, angiosarcomas of the nasal tract remain a diagnostic challenge due to their propensity for local invasion and distant metastasis. Early diagnosis can be helpful in the management of the disease. Further research is needed to optimize treatment approaches and can be helpful in the long-term survival of nasal angiosarcoma patients.
